# Astrocyte alterations in neurodegenerative pathologies and their modeling in human induced pluripotent stem cell platforms

**DOI:** 10.1007/s00018-019-03111-7

**Published:** 2019-04-23

**Authors:** Minna Oksanen, Sarka Lehtonen, Merja Jaronen, Gundars Goldsteins, Riikka H. Hämäläinen, Jari Koistinaho

**Affiliations:** 1 A.I.Virtanen Institute for Molecular Sciences, University of Eastern Finland, 70210 Kuopio, Finland; 2 Neuroscience Center, Helsinki Institute of Life Science, University of Helsinki, PO. Box 63, 00290 Helsinki, Finland

**Keywords:** Astrocytes, Neurodegeneration, Alzheimer’s disease, Parkinson’s disease, Amyotrophic lateral sclerosis

## Abstract

Astrocytes are the most abundant cell type in the brain. They were long considered only as passive support for neuronal cells. However, recent data have revealed many active roles for these cells both in maintenance of the normal physiological homeostasis in the brain as well as in neurodegeneration and disease. Moreover, human astrocytes have been found to be much more complex than their rodent counterparts, and to date, astrocytes are known to actively participate in a multitude of processes such as neurotransmitter uptake and recycling, gliotransmitter release, neuroenergetics, inflammation, modulation of synaptic activity, ionic balance, maintenance of the blood–brain barrier, and many other crucial functions of the brain. This review focuses on the role of astrocytes in human neurodegenerative disease and the potential of the novel stem cell-based platforms in modeling astrocytic functions in health and in disease.

## Introduction

Astrocytes are the most abundant non-neuronal cell type in the central nervous system (CNS). They serve numerous critical functions in the brain, including regulation of synapse formation and pruning, as well as regulation of neuroinflammation and lactate and glutamate levels in the brain [1]. Astrocytes play a key role also in several homeostatic and neuroprotective functions and are essential for the integrity of the blood–brain barrier (BBB). Primary astrocyte defects have been implicated to impact neurodegenerative diseases, and both a loss of their normal supportive function as well as a gain of toxic functions are thought to underlie the neuronal death in acute or chronic brain diseases [2, 3]. This review will address the main functions of astrocytes involved in neurodegeneration.

## Astrocytes in synaptic defects

Synaptic dysfunction and degeneration are common features in many neurodegenerative diseases, such as Alzheimer’s disease (AD), and amyotrophic lateral sclerosis (ALS). Synaptic defects establish in the early stages of the diseases and synapse loss is often considered a primary pathological feature and not just secondary to the typical neuron loss in these diseases.

### Astrocytes are part of the tripartite synapse

**Fig. 1:**
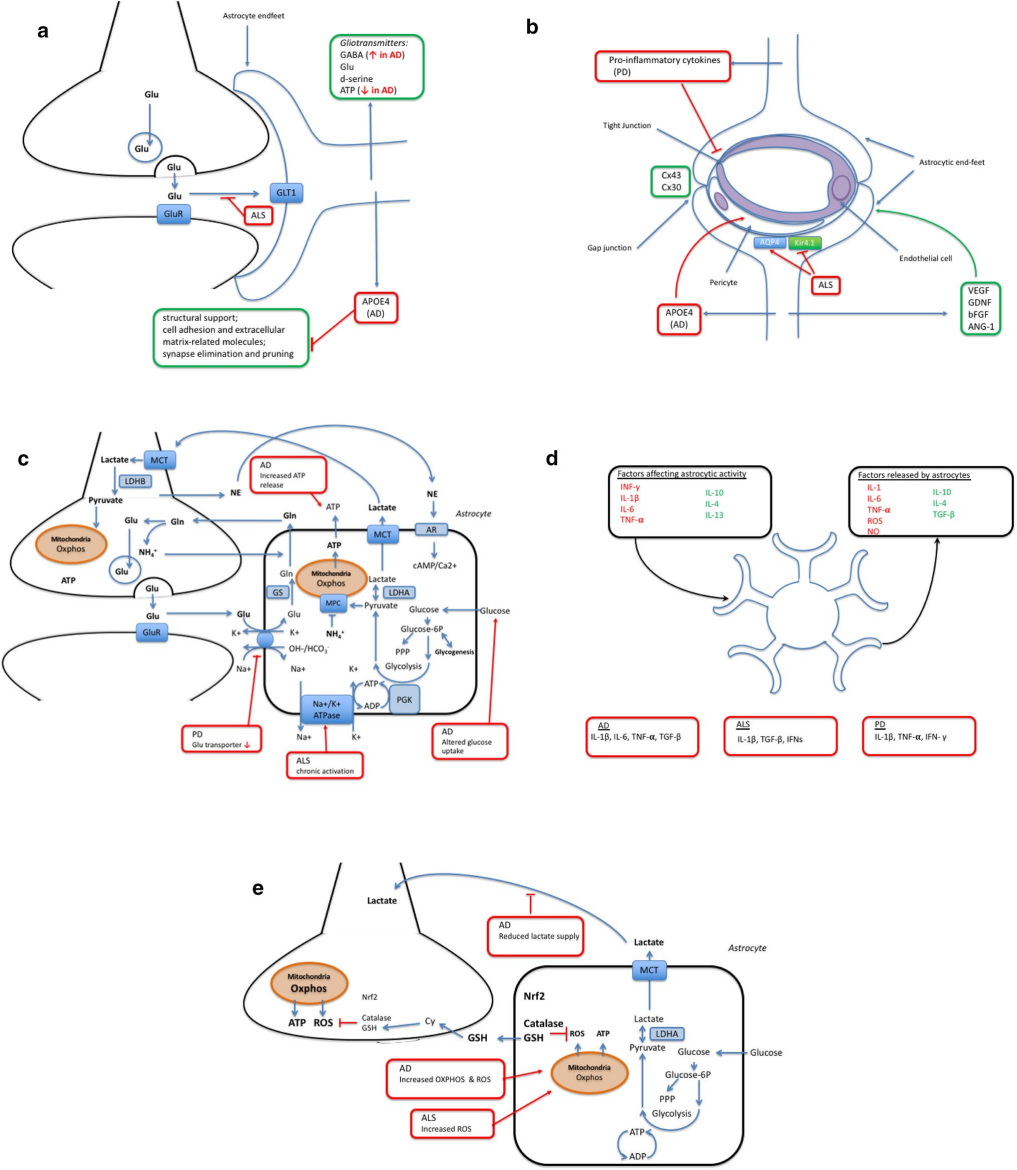
**a** Astrocytes and the pre- and post-synaptic endings of neurons form the tripartite synapse. Astrocytes clear neurotransmitters and secrete gliotransmitters, e.g., GABA, glutamate, d-serine, and ATP [5, 6]. In addition, they provide structural support and produce many cell adhesion and extracellular matrix-related molecules required for proper synaptic function. In AD, APOE4 astrocytes have decreased rate of synapse pruning and turnover in the brain [18]. In AD expression of GABA, the inhibitory gliotransmitter, in reactive astrocytes, is increased, whereas reduced other gliotransmitter release, especially ATP [22–24]. **b** Blood–brain barrier. Astrocytic perivascular endfeet express water channel aquaporin-4 (AQP4) and the ATP-sensitive inward rectifier potassium channel Kir4.1, and transporter proteins such as glucose transporter-1 and P-glycoprotein [37]. Astrocytes support the BBB through the release of several growth factors including vascular endothelial growth factor (VEGF), glial cell line-derived neurotrophic factor (GDNF), basic fibroblast growth factor (bFGF), and angiopoietin 1 (ANG-1) [41]. Astrocytes communicate with each other through gap junction proteins mainly connexin (Cx) 43 and Cx 30. Astrocytic APOE4 promotes BBB disruption in AD. The release of pro-inflammatory cytokines including IL-6, IL-1B, and TNF-A in PD increase neuronal death and rearrange TJ protein expression on ECs. Astrocyte ability to maintain water and potassium homeostasis is reduced in ALS due to increased expression of AQP4 and reduced Kir4.1 [75]. **c** Astrocytes provide metabolic support to the neurons and maintain neurotransmitter homeostasis, actively responding on neuronal signals [86]. In neurodegenerative disease, these functions are compromised. Astrocytes exposed to amyloid peptides alter glucose uptake and its downstream metabolism in parallel with increased hydroperoxide and glutathione release. AD impacts astrocytic metabolism causing increased glutamate and ATP release, what in turn results in stimulation of microglial activation and neuronal vulnerability [101]. Astrocytic expression of PD-related A53T mutant alpha-synuclein results in down regulation of glutamate transporters and wide-spread gliosis [108]. In ALS, chronic activation of the α2-Na/K ATPase/α-adducin complex in astrocytes has been demonstrated, resulting in biased energy metabolism and acquisition of pro-inflammatory phenotype [131]. **d** Astrocytes mediate inflammatory effects in neurodegeneration. Astrocytic activity is affected by plethora of cytokines; INF-γ, IL-1β, IL-6, and TNF-α lead to classical activation, whereas IL-10, IL-4, and IL-13 induce alternative activation with decreased ROS and NO production alleviating inflammation [139]. In addition, astrocytes release factors affecting their inflammatory environment. Different cytokine levels are known to be affected in neurodegenerative disorders. In AD, astrocyte associated inflammation has been linked to IL-1β, IL-6, TNF-α, and TGF-β leading to activation of microglial cells [145]. Whereas, levels of IL-1β, TGF-β, and IFNs have been demonstrated to be elevated in post-mortem tissues of ALS patients [161]. Finally, in PD, levels of IL-1β, TNF-α, and IFN- γ are shown to be affected [153, 154]. **e** Astrocytes and oxidative stress in neurodegenerative diseases. In normal physiological conditions, astrocytes are glycolytic, whereas neurons are more oxidative and produce much more ROS than astrocytes do; however, increased ROS production by astrocytes has been reported in both AD and ALS [187, 196]. On the other hand, astrocytes have much higher antioxidant capacity than neurons and express higher levels of Nrf2, catalase and GSH, than neurons. Oxidative stress induces GSH release from astrocytes, which is degraded, and the cysteine-rich degradation products are taken up by neurons and used for neuronal GSH synthesis. [198–200]

Astrocytes have critical roles in controlling synaptic development, functionality, plasticity, and elimination (reviewed in Ref. [4]). First of all, the presence of astrocytes is required for synapse formation, and glial depletion results in loss of synaptic connections followed by neurodegeneration. Astrocytes secrete synaptogenic factors, including thrombospondins, which regulate the number and formation of synapses [5, 6]. In addition to the secreted signaling molecules, a direct contact of neurons with astrocytes seems to be essential for synapse formation [6]. Second, astrocytes regulate the functionality of synapses in many ways. They contact and ensheath the pre- and post-synaptic endings of neurons in the human brain [7] and communicate with them to form the tripartite synapse [8] (Fig. 1a). Astrocytes fine-tune the strength of synaptic signals by regulating the pre-synaptic vesicle release and post-synaptic receptor composition through secreted molecules [4]. For example, astrocyte-secreted cholesterol has been shown to enhance pre-synaptic glutamatergic signaling [9]. Astrocytes also provide structural support and produce many cell adhesion and extracellular matrix-related molecules (e.g., neuroligins, cadherins, glypicans, and laminins) required for proper synapse formation and function (reviewed in Ref. [10]). In addition, astrocytes and astrocyte-secreted factors play a role in the different aspects of neuronal circuits and synaptic plasticity, including long-term potentiation, long-term depression, and synaptic scaling. Astrocytes are capable of regulating and clearing neurotransmitters in the synaptic cleft, but they also communicate by secreting the so-called gliotransmitters (e.g., GABA, glutamate, d-serine, and ATP) as a result of increased intracellular Ca^2+^ levels. These gliotransmitters act on various excitatory and inhibitory targets and are known to modulate synaptic transmission and plasticity (reviewed in Ref. [11]). The recent finding that transplantation of human astrocytes significantly improves the cognitive behavior of mice further supports the role of astrocytes in synaptic plasticity and cognition [12]. Finally, astrocytes have been shown to participate in synapse elimination and pruning, and thus refinement of neuronal circuits, through phagocytosis [13]. This process is regulated by two phagocytic receptor pathways, multiple EGF-like domains 10 (MEGF10) and c-mer proto-oncogene tyrosine kinase (MERTK) at least in mice. Taken together, astrocytes are constantly monitoring and regulating synapses in many ways through direct contact and various signaling molecules, and thus, they are likely to play an essential role in synaptic defects linked to neurodegenerative disorders.

### Astrocytes are linked to synaptic defects in neurodegenerative diseases

The best evidence for synaptic defects in neurodegenerative diseases comes from AD studies. In AD, synapse dysfunction is one of the most striking early pathological features. There is a progressive loss of synapses during the disease course, and in fact, the extent of synapse loss is the best correlate with cognitive decline [14]. The exact mechanisms behind the synapse loss and synaptic defects still remain unclear, although several theories involving beta-amyloid (Aβ) plaques, complement cascade pathway, and microglia have been proposed [15] [16]. In addition, the importance of astrocytes in AD and synaptic defects is currently strongly emerging and this review will address some of the recent advances supporting it. For example, astrocyte-derived cholesterol has been shown to be a crucial factor in promoting synaptogenesis [9]. Apolipoprotein E (apoE), the major cholesterol carrier in the brain, is mainly synthesized and secreted by astrocytes. APOE polymorphisms are the main genetic determinants for AD with the APOE4 allele being associated with increased AD risk [17]. In mouse studies, APOE4 astrocytes showed decreased rate of synapse pruning and turnover in the brain, leading to the accumulation of senescent, non-functional synapses, when compared to APOE3 astrocytes [18]. This could be linked to the increased synaptic vulnerability in AD. Zhao and colleagues used human iPSC-derived neurons and astrocytes in a transwell-based co-culture system to study the effects of APOE4 astrocytes on control neurons [19]. They found that APOE4 astrocytes were less capable of promoting synapse formation and the expression of synaptic proteins was decreased in comparison with APOE3 astrocytes. Furthermore, APOE4 astrocytes reduced the survival of neurons and eventually led to neurodegeneration. These data support the importance of astrocytes and APOE genotype in synaptic deficiencies and neurodegeneration linked to AD. Another astrocyte-secreted synaptogenic molecule, thrombospondin-1 (TSP-1), has decreased expression level in the brain of AD patients [20]. On the other hand, treatment with TSP-1 was shown to reverse the Aβ-induced synaptic dysfunction in vivo.

Intriguingly, astrocytes were very recently found to engulf and phagocytose dystrophic neurons and pre-synaptic elements associated with amyloid plaques in APP/PS1 mutant AD mice as well as in post-mortem human brains [21]. This result supports the role of astrocytes in synaptic regulation and phagocytosis also in the human brain, although the exact meaning for disease pathogenesis remains to be elucidated. It is hypothesized that the astrocytic clearance of dystrophic neurites and dysfunctional synapses is inadequate in AD, resulting in their accumulation and impaired neural networks. In mice, the rate of synapse pruning decreases upon aging leading to the accumulation of senescent synapses, a possible explanation behind the cognitive decline [4, 13].

Reactive astrocytes in post-mortem AD patient’s brain show markedly increased expression of GABA, the inhibitory gliotransmitter. The enhanced GABA signaling has been linked with memory and learning deficits in mouse models of AD [22]. In addition, a specific type of reactive astrocytes shows diminished potential to promote synaptogenesis and synaptic function, leading to a decreased number of synapses with only weak signals and, finally, neurodegeneration [23]. This kind of reactive astrocytes seems to be abundant in post-mortem AD brain. In mouse primary hippocampal cultures, astrocytes internalized oligomeric tau, which was followed by reduced gliotransmitter release, especially ATP [24]. The reduced ATP release from astrocytes reduced the synaptic transmission of neurons and expression of both pre- and post-synaptic proteins in them.

In other neurodegenerative diseases, there is so far less evidence supporting the role of astrocytes in synaptic defects. In ALS, astrocytes have a well-established role in the degeneration of motor neurons and astrocytes with both sporadic (sALS) and familial (fALS) background seem to have similar effects when studied either in vitro or in vivo [25–28]. ALS is characterized by synaptic disconnections at the neuromuscular junction leading to axonal degeneration and, finally, death of neurons through either cell-autonomous or non-cell-autonomous mechanisms [29]. When transplanted into the mouse spinal cord, human sALS astrocytes have been shown to lead to reduced pre-synaptic connections and denervation at the neuromuscular junctions [28]. There is also a significant loss and degeneration of synapses in the frontal cortex in ALS, but this has not been clearly linked to astrocytes or astrogliosis in the post-mortem human brain [30].

In synucleinopathies such as Parkinson’ disease (PD) and dementia with Lewy bodies (DLB), a pre-synaptic protein α-synuclein aggregates to form insoluble fibrils. Recently, numerous small deposits of α-synuclein aggregates that are not associated with Lewy bodies were demonstrated at the presynapses in PD DLB cases. Moreover, dendritic spines showed retraction, whereas the presynapses were mostly preserved, suggesting a neurotransmitter deprivation [31]. As α-synuclein released from neuronal cells can be taken up by astrocytes and induce inflammatory response and reduced release of neurotrophic factors, astrocytes may regulate synaptic functions related to pre-synaptic deposition of α-synuclein (Table 1).

**Table 1:** Astrocyte dysfunctions in neurodegenerative pathologies

Neurodegenerative pathology	Disease	Pathological hallmark/mechanism proposed	References
1. Synaptic pathology	AD	Decreased rate of synapse pruning	[18]
		Increased expression of GABA	[22]
		Reduced gliotransmitter ATP release	[24]
	PD	α-Synuclein aggregates in the synaptic terminals	[31]
	ALS	Reduced pre-synaptic connections and NMJ denervation	[28]
2. BBB dysfunction	AD	BBB leakage due to loss of tight junction barrier function	[61, 63, 64]
		Endfeet retraction and reduced expression of GLUT1 and lactate transporters	[54]
		AQP4 deficient mice are unable to clear soluble Aβ	[38]
		Astrocytic APOE4 accelerates pericyte loss and microvascular reduction	[57]
	PD	Increased permeability of the BBB associated with decreased expression of P-glycoprotein	[65, 66]
	ALS	BBB disturbances in both sALS and patients with SOD1 mutation	[71]
		Increased AQP4-promoted perivascular edema-linked BBB opening	[74, 75]
3. Metabolic dysfunction	AD	Triggering amyloid beta deposition	[97] [188]
		Metabolically regulation of beta and gamma secretase levels	[98, 99]
		Aberrant post-translational modification of APP	[100]
		Altered glucose uptake and metabolism in parallel with increased hydroperoxide and glutathione release	[101]
		Increased glutamate and ATP release through connexin 43 hemichannels	[102, 103]
	PD	Astrocytic expression of mutant alpha-synuclein resulted in down regulation of glutamate transporters	[108]
	ALS	Impaired supportive capacity to motor neurons	[117, 118]
		Disruption of the TCA cycle and glutamate metabolism	[126]
		Dysregulation of purine, pyrimidine, lysine, and glycerophospholipid metabolism pathways	[127]
		Impairment of the astrocyte lactate transport and pro-nerve growth factor-p75 receptor signaling	[129]
		Chronic activation of the α2-Na/K ATPase/α-adducin complex, resulting in biased energy metabolism	[131]
4. Oxidative stress	AD	Increased ROS due to activation of astrocytic NADPH oxidase	[186]
		Activated PARP, leading to decreased NAD levels and metabolic failure	[187]
		Increased ROS and Ca^2+^ leakage from ER leading to opening of the mitochondrial permeability transition pore	[190]
		PSEN1 mutant astrocytes manifest Aβ-deposits and altered metabolism with increased ROS production	[188]
	ALS	Increased ROS production which can be prevented by antioxidants	[196]
5. Neuroinflammation	AD	Release chemokines, cytokines, and increase the production of Aβ in response	[142]
		Hypertrophic reactive astrocytes accumulate around the Aβ plaques	[143]
		Increase in reactive A1 astrocytes	[23]
		MAO-B highly expressed in astrocytes surrounding amyloid plaques	[239]
	PD	Presence of reactive astrocytes in the substantia nigra pars compacta	[148]
		Genes implicated in PD pathology play a role in the astrocyte activation in response to inflammatory stimuli	[149–152]
	ALS	Pro-inflammatory mediators upregulated in ALS-patient tissues and stem-cell-derived human astrocytes	[113, 158–160]
		A strong astrocytic A1-phenotype in the motor cortex of ALS patients	[23]

In Huntington’s disease patients, reduced glutamate uptake has been detected and this is linked to astrocytes, since glutamate is cleared from the perisynaptic area mostly by astrocytes [32]. In Down’s syndrome, there is reduced level of astrocyte-secreted TSP-1 leading to decreased synaptic density and spine malformations [33]. Taken together, these findings support the importance of astrocytes, astrocyte-secreted gliotransmitters, and other factors in synapse defects and neurodegeneration, although more human studies are needed for confirmation.

## Astrocytes in the maintenance of the blood–brain barrier in neurodegenerative diseases

The blood–brain barrier (BBB) is a semipermeable membrane that separates the circulating blood from the CNS. It regulates delivery of oxygen and important nutrients to the brain through active and passive transport and prevents neurotoxins from entering the brain. It also has a clearance function and removes carbon dioxide and toxic metabolites from the CNS. A well-functioning BBB is essential for maintaining healthy brain tissue, and BBB breakdown or dysfunction is associated with a variety of neurological diseases due to the accumulation of blood-derived neurotoxins in the CNS that causes progressive neurodegeneration [34]. Although the most important components of the BBB are endothelial cells (ECs), astrocytes contribute to BBB stability through their direct contact of perivascular endfeet with ECs and pericytes (Fig. 1b).

Astrocytes through their endfeet establish the link between the endothelial cells and neurons and are important in the development and maintenance of BBB. Astrocytic perivascular endfeet surround ~ 98% of the basal parenchymal membrane of brain microvessels and express molecules which regulate ionic concentrations and protein transporters in the BBB [35, 36]. Among the most abundant ones are water channel aquaporin-4 (AQP4) and the ATP-sensitive inward rectifier potassium channel Kir4.1, as well as transporter proteins such as glucose transporter-1 and P-glycoprotein, indicating the importance of the endfeet polarization for proper functioning of BBB [37]. Interestingly, the location of AQP4 follows the distribution of inwardly rectifying potassium channel 4.1 (Kir4.1) [38], and together, they control water balance and ion homeostasis at the level of BBB and neuronal synapses [39, 40]. Furthermore, astrocytes support the BBB maintenance through the release of several growth factors, including vascular endothelial growth factor (VEGF), glial cell line-derived neurotrophic factor (GDNF), basic fibroblast growth factor (bFGF), and angiopoietin 1 (ANG-1) [41]. The upregulation of ANG-1 leads to enhanced barrier tightness through increased expression of junctional proteins, such as occludin and claudins and inhibition of leukocyte transmigration after TNFα stimulation. The identification of astrocyte-secreted factors suggests that mature astrocytes rather modulate and maintain the barrier function than induce its cerebrovascular integrity, because astrocytes initially appear at the neurovascular unit (NVU) postnatally [42]. Astrocytes regulate the blood flow according to demands of neuronal synaptic activity [43] by elevating Ca^2+^ levels in the endfeet [44]. Depending on released mediators such as prostaglandin E, nitric oxide, or arachidonic acid from astrocytes, blood vessels in brain constrict or dilate [45, 46]. Astrocytes also produce a cholesterol and phospholipid transporter molecule APOE, which mediates regulatory processes related to brain homeostasis [47]. Adult ApoE knockout mice show increased permeability selectively of cerebral vessels and leakage of serum proteins into the CNS tissue [48]. Whereas APOE3, the most abundant human APOE isoform, and APOE2 mediate physiological BBB tightness, APOE4 promotes BBB disruption, as observed in mutant mice in which mouse Apoe was replaced by human APOE isoforms [49]. Thus, homeostasis of the neurovascular junction is fundamental for cognitive functions, and homeostatic imbalances may be linked to cognitive changes.

### Astrocytes in BBB dysfunction

Reactive astrogliosis is a common feature of astrocytes and compromises neuronal survival. This process involves both molecular and morphological changes in astrocytes and highly depends on the events triggering it. Therefore, it may have both beneficial and detrimental effects on surrounding cells [50]. Astrocytes communicate with each other through gap junction proteins, mainly connexin (Cx) 43 and Cx 30. Mice lacking Cx43 and Cx30 expression on astrocyte endfeet manifest a significant loss of AQP4 and β-dystroglycan, a transmembrane receptor anchoring astrocyte endfeet to the perivascular basal lamina. The absence of astroglial connexins weakens the BBB that opens upon increased hydrostatic vascular pressure and shear stress [51].

### Alzheimer’s disease

Accumulating data support the importance of vascular astrocytes in AD pathology [52, 53]. Astrocyte endfeet surrounding vascular Aβ deposits showed morphological changes including retraction and swelling, and reduced expression of GLUT1 and lactate transporters in transgenic arctic β-amyloid (arcAβ) mice expressing human amyloid precursor protein (APP) [54]. These changes occur at early stages of the disease and are consistent with NVU uncoupling, suggesting that astrocyte dysfunction contributes to the early behavioral and cognitive impairments. AQP4 deficient mice are unable to clear soluble Aβ efficiently, proposing that this pathway may remove Aβ from the CNS [38]. In humans, the loss of perivascular AQP4 localization was associated with increased Aβ burden and increased AD pathology (Braak stage) [55]. Furthermore, primary human astrocytes obtained from non-demented human brain tissue upregulate the expression of neprilysin and scavenger receptor B1 as a response to Aβ–APOE complexes. This function appears to be defective in astrocytes derived from AD brain [56]. APOE4, which in CNS is mainly secreted by astrocytes, accelerates pericyte loss and microvascular reduction by enhancing pro-inflammatory pathway both in pericytes and ECs [57]. These findings suggest that Aβ clearance of astrocytes is impaired in AD due to astrocyte polarization induced by brain Aβ deposition [58, 59]. Nevertheless, the contribution of Aβ-induced changes to BBB is still unclear.

AD patients’ brains are characterized by high prevalence of degenerated endothelium and impairment of BBB transport system [34, 60]. BBB breakdown in the hippocampus precedes neurodegeneration [61, 62] and follow-up studies in AD patients have confirmed BBB leakage in both gray and white matter regions due to a loss of tight junction barrier function [61, 63, 64]. Moreover, cognitive decline was associated with stronger BBB leakage in patients with an early AD, suggesting that BBB damage might be part of a cascade of pathologic events that eventually leads to dementia [61]. In aging brain, altered AQP4 expression and its redistribution from astrocytic endfoot membranes to non-end-foot membranes compromise Aβ clearance through the glymphatic system and likely contribute to AD pathology [55].

### Parkinson’s disease

Initially, it was assumed that the BBB stays unaltered during the development of PD, but several studies with human and animal models have linked BBB dysfunction with the development of PD pathology. In studies with PD patients, the increased permeability of the BBB was associated with decreased expression of P-glycoprotein demonstrated by increased uptake of [^11^C]-verapamil in the midbrain [65] and frontal white matter regions in PD patients [66]. Taking into account that P-glycoprotein is an active drug transporter, it is possible that the reduction of P-glycoprotein may be associated with accumulation of neurotoxins and aggregated proteins in the PD brain affecting directly dopaminergic neuron survival [67]. In addition to human studies, several toxin-induced animal models, including 6-OHDA treated rats and MPTP-treated mice, have shown a disruption of the BBB that is linked to the loss of dopaminergic neurons [68, 69]. It has also been shown that activation of microglia and astrocytes in PD leads to the release of pro-inflammatory cytokines, including IL-6, IL-1B, and TNFα, which can increase neuronal death and rearrange tight junction protein expression on ECs [70]. Moreover, the loss of signaling interaction between astrocytic perivascular endfeet and ECs leads to hypertrophy and upregulation of GFAP and vimentin in astrocytes triggering reactive astrogliosis and weakens BBB tightness [41].

### Amyotrophic lateral sclerosis

In ALS, neurodegeneration of upper and lower motor neurons in the brain and spinal cord is not completely understood. The pathogenesis of the disease is complex with recent evidence showing BBB disturbances in the brain of fALS patients that carry SOD1 mutation and in post-mortem brains of sALS patients [71]. Primary degeneration of ECs and pericytes compromising vascular barrier integrity both in the brain and spinal cord of ALS patients has been described. The BBB disruption could be the main obstacle for efficient drug delivery to the CNS due to the impairment of P-glycoprotein efflux transporter in the brain and spinal cord microvessels [72, 73]. Therefore, restoration of BBB integrity could promote delivery of therapeutics to the CNS as well as help to remove waste products from the brain, providing an improved environment for motor neuron survival. The astrocyte contribution to BBB disruption in ALS has been studied only in rodents. In end-stage SOD1, rats increased expression of AQP4 in the spinal cord gray matter and around blood vessels promoted perivascular edema-linked BBB opening in ALS [74]. As elevated AQP4 was also detected near motor neurons, it is possible that dysfunctional astrocytes contribute to further motor neuron degeneration [75]. In parallel Kir4.1 expression was decreased in the brainstem and cortex of SOD1 rats. Thus, ability of astrocytes to properly maintain water and potassium homeostasis in CNS is reduced, not only affecting the BBB but also impeding motor neuron survival in ALS. In pre-symptomatic SOD1 mutant mice, BBB breakdown has been demonstrated prior to motor neuron degeneration and inflammatory changes in the spinal cord [76]. Whether connexin-mediated changes are directly affecting BBB function in ALS, is not known yet. However, although BBB damage might not be a causative event in ALS, it is still a significant, and possibly critical, pathologic effector for the disease progression.

### In vitro models of the BBB

To better understand the role of human astrocytes in BBB functionality in neurodegenerative diseases and to develop new treatment strategies with predictive drug permeability, there is a demand for development of improved BBB models which include astrocytes and other components of human origin. Current in vitro BBB models mostly use brain microvessels and astrocytes of bovine, porcine, or rodent origin [77]. Even though the models are well established, the characteristics and functionality of the BBB in these species differ from that in humans especially in the expression level of important transporters such as P-glycoprotein [78–81]. The first human BBB models were generated from primary and immortalized human cells [82, 83]. However, the limited availability of primary human cells and lack of barrier tightness in immortalized cells underlines the need for more accessible and relevant cell source for BBB modeling. Recent advances in stem cell technology have created the opportunity to generate cells important for building human in vitro BBB [84, 85]. Stem cell sources have demonstrated a significant advantage over other cell types in respect of human origin, scalability, self-renewal, and potential to generate all BBB cell components from the same patient.

## Metabolic dysfunction of astrocytes in neurodegenerative diseases

Astrocytes are actively participating in many metabolic functions of the CNS. Even though for the time being none of the human neurodegenerative diseases is known to originate from the astrocytic cell-autonomous dysfunction, astrocyte–neuronal metabolic coupling plays a central role in the physiological CNS homeostasis (Fig. 1c). The role of astrocytes spans far beyond the participation in a tripartite synapse and astrocytes have an impact on virtually every function classically attributed to neurons. Moreover, astrocytes provide metabolic and structural support to neuronal circuits. A recently discovered ability of astrocytes to regulate the respiratory rhythm-generating neuronal circuits through mechanism involving vesicular release indicates a key role of astrocytes in modulation of CNS activities [86].

The metabolic demand of CNS is very high, comprising approximately ten times over the average for all tissues. The major metabolic consumers here are the functions needed for maintenance of the membrane potential and ion transport across the membranes. On the other hand, the brain contains little substances for energy production and relays mainly on supplies from the circulation [87]. The main energy substrate for the CNS function is glucose, in parallel with ketone bodies which complement energy source upon fasting [88]. Astrocytes are thought to be the main glucose utilizing cells converting it to lactate and pyruvate and creating glycogen deposits. Astrocytes are also capable of ketogenesis, which besides providing an alternative source for energy metabolism may also protect against accumulation of non-esterified fatty acids and prevent formation of pro-apoptotic ceramide [89]. Providing anatomically a link between circulation and neuronal circuits, astrocytes serve as a major regulator of neuronal metabolism. All these activities are coupled and orchestrated with neuronal activity. Thus, astrocytes also support neurons by delivering substrates for energy metabolism, biosynthesis and neurotransmission, as well as take care of waste processing.

Even though neurons and astrocytes have an equal capacity to fully oxidize both glucose and lactate, they demonstrate physiological preferences resulting in astrocytes being mostly glycolytic in contrast to neurons that favor oxidative phosphorylation. There may be several reasons determining such metabolic specialization. First off, compared to astrocytes neurons have higher energy need, which can be met by oxidative metabolism. Second, neurons may favor glucose utilization through pentose phosphate pathway (PPP) to maintain sufficient NADPH levels and antioxidant potential. Upon these circumstances, neuronal lactate oxidation allows a support to sufficient mitochondrial ATP production without increasing glycolytic activity [90].

Astrocytes are indispensable for supporting both GABAergic and glutamatergic neurotransmission in brain. The available pool of these neurotransmitters depends on the glutamine synthesis in astrocytes [91]. In contrast to neurons, astrocytes express pyruvate carboxylase and are able to replenish their citric acid cycle intermediates carboxylating pyruvate to oxaloacetate, what is known as anaplerosis [88] [92].

The major waste produced by neuronal activity are carbon dioxide and ammonia. While carbon dioxide diffuses across BBB, ammonia is taken up by astrocytes and then released to neurons [92]. Ammonia enters astrocytes through potassium channels and transporters and being processed is released as glutamine [93].

In the adult brain, glycogen is deposited in astrocytes [94]. Glycogenolysis and the lactate produced in astrocytes play a critical role in long-term memory formation [95]. It has been demonstrated that pharmacological inhibition of either astrocytic glycogenolysis or monocarboxylate transporter responsible for neuronal lactate uptake impair memory and this impairment can be reversed by administration of lactate or glucose [96]. Thus, compromised astrocyte metabolic functions may result in neuronal dysfunction and lead to the development of neurodegeneration. Here, we will focus on the most relevant metabolic aspects of astrocytes having importance for neurodegenerative diseases.

### Alzheimer’s disease

Astrocytic metabolic dysfunction has been suggested be a triggering factor for amyloid beta (Aβ) deposition [97]. While amyloid deposition has been mostly seen as a consequence of reduced Aβ clearance, increased production of amyloidogenic species may take place through metabolic regulation of beta and gamma secretase levels [98, 99], or aberrant post-translational modification of amyloid precursor protein APP [100]. It has been demonstrated that exposure of cultured astrocytes to amyloid peptides alters their glucose uptake and its downstream metabolism in parallel with increased hydroperoxide and glutathione release [101]. The pro-inflammatory environment typically present in AD brain may alter astrocytic metabolism causing increased glutamate and ATP release through connexin 43 hemichannels, which in turn could result in microglial activation and neuronal vulnerability [102, 103].

### Parkinson’s disease and Huntington’s disease

The degenerating brain areas in PD, such as SN, contain relatively less astrocytes than other brain areas, making dopaminergic neurons potentially more vulnerable to the metabolic and energy disturbances [104]. In line with this assumption, it has been demonstrated that glucose deprivation promotes α-synuclein aggregation [105], whereas increased lactate supply inhibits it [106], providing a rationale for involvement of astrocytic metabolic dysfunction in PD pathogenesis. Moreover, astrocytes isolated from PD animal models expressing mutant α-synuclein or deficient for Parkin showed functional and morphological deficits of mitochondria, which was linked to astrocytes’ inability to support neuronal differentiation [107]. In addition, expression of PD-related A53T mutant α-synuclein in astrocytes was found to result in downregulation of glutamate transporters and wide-spread gliosis [108]. In the context of PD treatment, astrocytes have been shown to convert l-DOPA to dopamine [109], and in the striatum, astrocytes serve for l-DOPA storage [110]. It is, therefore, likely that metabolic and energy disturbances in astrocytes contribute to not only development of PD, but also in deteriorating treatment responses.

Several lines of research indicate astrocytic contribution also in HD. In a mouse model of HD, a deficit of astrocytic GABA release through GAT-3 has been thought to cause reduced tonic inhibition neuronal output in the striatum [111].

Furthermore, in HD mouse models astrocytes have shown increased Ca^2+^-dependent exocytotic glutamate release which correlates with elevated biosynthesis of glutamate due to upregulation of astrocytic pyruvate carboxylase [112].

### Amyotrophic lateral sclerosis

Both non-cell-autonomous and cell-autonomous mechanisms are implicated in glial cell contribution to ALS [113–115], and astrocytes appear to be major determinants of ALS progression. Animal studies indicate a toxic gain of function in ALS astrocytes, which may converge into impaired supportive capacity of astrocytes to motoneurons [26, 116]. Subsequently, the initial astrocyte cell-autonomous dysfunction may lead to the non-cell-autonomous neuronal toxicity [117, 118].

Application of metabolomics, transcriptomics, and lipidomics on ALS models indicates significant dysregulation of a number of metabolic pathways in ALS astrocytes [119–125]. Metabolomics studies on models composed from motoneurons and astrocytes expressing mutant human SOD1 revealed disruption of the TCA cycle and glutamate metabolism under oxidative stress induced by menadione [126]. Furthermore, dysregulation of purine, pyrimidine, lysine, and glycerophospholipid metabolism pathways was demonstrated in ALS astrocytes co-cultured with motoneurons. In this model, glutamate exposure caused significant alterations in the astrocyte metabolic fingerprint and associated with decreased lactate levels by affecting lactate efflux from astrocytes [127]. Moreover, these findings correlate with the data from CSF analyses of human ALS patients [120]. Importantly, ALS in human patients has been associated with hypermetabolism, possibly involving aberrant astrocytic mitochondrial metabolism [128], which is present already at the pre-symptomatic state in SOD1 mutant mouse models and may thus have a key role of in early disease pathogenesis [129].

Analysis of ALS models expressing mutant SOD1 reveals prominent astrocytic alterations linked to metabolic dysregulation due to impairment of the astrocyte lactate transport and pro-nerve growth factor-p75 receptor signaling [129]. In addition, aberrant activation of metabotropic receptor 5 mGluR5 has been demonstrated in ALS astrocytes causing persistent elevation of intracellular calcium [130]. More recently, a specific chronic activation of the α2-Na/K ATPase/α-adducin complex in ALS astrocytes has been demonstrated, resulting in biased energy metabolism and acquisition of pro-inflammatory phenotype [131].

It has been demonstrated that astrocytes also potentially regulate neuronal proteostasis by affecting autophagic degradation pathways. The ability of conditioned medium from ALS-patient iPSC-derived astrocytes to modulate the autophagy pathway neuronal cells has been linked to the decreased expression of LC3-II and concomitant increase in the expression of SOD1 [132].

Overall, the current research demonstrates that astrocytic non-cell-autonomous and cell-autonomous mechanisms including metabolic dysfunction and related changes in gene expression play a central role in pathogenesis of neurodegenerative diseases, and identification of molecular targets in astrocytes can serve for designing therapy that could prevent or slow down the disease progression.

## Astrocytes in the inflammation associated with neurodegeneration

Although the exact mechanisms of neurodegeneration are not known, recent data have identified the inflammatory processes tightly linked to multiple neurodegenerative pathways [133]. Regardless of the immune reaction within the CNS, it always involves astrocytes and microglia [134], the resident immune cells of the CNS. Under physiological conditions, astrocytes are key cells for the maintenance of homeostatic balance [135]. However, under pathological inflammatory state, they may produce neurotoxic factors that amplify the disease state, indicating the persistence of inflammatory stimuli or failure in normal resolution mechanisms [136]. This can result in the production of neurotoxic mediators, such as cytokines and interleukins [137], which are associated with several neurodegenerative diseases such as AD, PD, and ALS. The inflammation associated with these diseases is not typically the initiating factor of neurodegeneration. However, the emerging evidence on the sustained inflammatory response associated with the contribution of astrocytes in disease progression suggests contributory role for neuroinflammation in neuronal dysfunction and death.

Traditionally, increased GFAP expression has been considered as a hallmark of astrocyte activation in neurodegenerative disorders [138]. In addition, increased levels of pro-inflammatory cytokines such as INF-γ, IL-1β, IL-6, and TNFα are known to result in classical astrocyte activation, leading to increased activation of NF-κB pathway, production of ROS and NO, and further release of IL-1β, IL-6, and TNFα. On the contrary, augmented levels of IL-4 and IL-13 induce an alternative activation with increased secretion of IL-4 and decreased production of ROS and NO (Fig. 1d). However, astrocyte deactivation, including reduced immune surveillance and pro-inflammatory signaling, is mediated through high levels of IL-10 and TGF-β [139].

### Alzheimer’s disease

Neuroinflammation is a major contributor to AD, driven by the activation of microglia and astrocytes. A recent study of CSF markers from several hundred individuals with mild cognitive impairment and patients with AD dementia revealed increased levels of YKL-40, ICAM-1, VCAM-1, IL-15, and Flt-1 during the pre-clinical, prodromal, and dementia stages of AD [140]. There was a correlation of these biomarkers with increased CSF levels of total tau, especially for YKL-40, a glycoprotein secreted by microglia and astrocytes. Interestingly, high astrocytic expression of IL-15 has also been shown to promote tissue damage in multiple sclerosis and following brain ischemia [141].

Aβ, the main pathological hallmark of AD, activates astrocytes to release chemokines and cytokines, and in an interaction with the increased levels of pro-inflammatory cytokines even increases the production of Aβ in neurons and astrocytes [142]. In fact, hypertrophic reactive astrocytes are strongly associated with Aβ pathology, as they accumulate around Aβ plaques, and are seen [143]. Especially, the pro-inflammatory A1 phenotype of astrocytes is seen in post-mortem tissue from AD patients. While Aβ has been demonstrated to induce a pro-inflammatory profile, and even astrogliosis in AD, it is not clear whether it is Aβ that induces this phenotype [23] [144].

In AD, neuroinflammation is characterized by the accumulation of cytokines such as IL-1β, IL-6, TNF-α, or TGF-β, which can, through activation of β- and γ-secretase activity, further contribute to cerebral amyloid deposition, augmentation of APP expression, Aβ formation, and finally to recruitment and activation of microglial cells [145].

Pro-inflammatory astrocyte activation appears to be linked to the release of synaptotoxic factors and loss of glutamate regulation. Dysregulation of Ca^2+^ homeostasis is thought to play the central role in these events. Due to the complexity of the Ca^2+^ balancing mechanisms, any malfunction in the regulatory checkpoints may contribute to the overall pathological alterations in neurodegenerative diseases. The Ca^2+^/calmodulin-dependent protein phosphatase, calcineurin has been suggested to provide a l link between Ca^2+^ dysregulation and astrocyte activation [146]. Calcineurin is highly expressed by subsets of activated astrocytes in both humans and animal models, resulting in the consecutive NFAT activation and contributing to the AD neuropathology [147].

### Parkinson’s disease

One of the key aspects in PD pathophysiology is neuroinflammation, including presence of reactive astrocytes, in the substantia nigra pars compacta [148]. This is thought to be a downstream response to the death of dopaminergic neurons, even though there is also increasing amount of evidence, suggesting that astrocytes have an initiating role in PD pathophysiology [149].

Several genes implicated in PD pathology, including SNCA, DJ-1, PLA2G6, ATP13A2, LRRK2, PINK, and Parkin, have been demonstrated to play a role in the origination and regulation of astrocyte activation in response to inflammatory stimuli [149]. Both α-synuclein and DJ-1 have been shown to affect the astrocytic TLR4 signaling [150–152]. Furthermore, if looking at the secreted pro-inflammatory mediators, Parkin expression is known to be modified by IL-1β and TNF-α stimulation [153] and DJ-1 has been shown to regulate astrocyte activation by IFN-γ  [154]. Finally, functional ATP13A2 expression prevents NLRP3 inflammasome activation [155].

Interestingly, a recent study demonstrated that post-mortem brain samples from PD patients display increased astrocytic senescence. Furthermore, paraquat, a known chemical to cause parkinsonism, induced astrocytic senescence and senescence-associated secretory phenotype with robust secretion of numerous inflammatory cytokines, chemokines, growth factors, and proteases both in in vitro and in in vivo settings [156]. Another recent report studying transplantation of neural progenitors in PD utilized co-transplantation of rat astrocytes and showed increased anti-inflammatory as well as decreased pro-inflammatory profile associated with behavioral improvement in the animals [235]. This further emphasizes the dual role of astrocytes in the neurodegeneration as astrocytes can either support the neurons or cause their degeneration.

### Amyotrophic lateral sclerosis

ALS is considered to be a non-cell-autonomous disease with substantial involvement of astrocytes [157]. Post-mortem studies of ALS patients have revealed increased GFAP expression in the motor cortex, motor nuclei of the brain stem, the corticospinal tract, and the ventral horn of the spinal tract [158]. Furthermore, astrocytic production of pro-inflammatory mediators, such as type 1 interferons, TGF-β1, and prostaglandin D_2_, has been demonstrated to be upregulated in ALS-patient post-mortem tissues and in ALS models based on embryonic stem cell-derived human astrocytes [113, 159, 160]. In addition, the astrocytic NLRP3 inflammasome expression has been shown to be increased, leading to augmented IL-1β production in the post-mortem tissues of sALS patients [161]. Most recently, the advances in the patient neuroimaging have allowed direct visualization of neuroinflammation in ALS. Although in the post-mortem tissues from ALS patients, astrocytosis is seen in parts of the corticospinal tract other than pons [162], PET imaging with [11C]-DED, a commonly used marker of activated astrocytes, was able to demonstrate increased uptake rate in pons and white matter [163], which may reflect corticospinal tract astrocytosis. Finally, a strong astrocytic A1-phenotype has been discovered in the motor cortex of ALS patients, further highlighting the pro-inflammatory phenotype of astrocytes in the areas affected by ALS [23].

### Huntington’s disease

Increased susceptibility to pro-inflammatory stimulation has been demonstrated in several mouse models of HD [164].

The enhanced inflammatory response in the brain was observed only in models with expression of mHTT including glial cells. The studies on primary astrocytes revealed increased IkB kinase activity, resulting in prolongation of NF-kB activation and upregulating pro-inflammatory factors, particularly TNF-α. Importantly, enhanced activation NF-kB-p65 was observed in astrocytes of patients and mice with HD [164]. Further attempts to block soluble TNF-α signaling using a dominant negative inhibitor XPro1595 caused decrease of TNF-α in the cortex and striatum, resulting in increased neuronal density and improved motor function. In addition, diminished amount of mutant HTT aggregates, and decreased gliosis was evident in brains of R6/2 mice [165].

## Astrocytes and oxidative stress in neurodegenerative diseases

Brain metabolism uses vast amounts of oxygen, and thus also produces free oxygen radicals that may damage the cells. At the same time, brain has relatively low antioxidant defense mechanisms and high incidence of peroxidation substrates, such as enrichment of unsaturated fatty acids and redox active transition metals [166]. Thus, the brain is one of the most susceptible tissues for oxidative damage. Oxidative stress plays an important role in various neurodegenerative disorders, in which age is one of the main risk factors and cumulative oxidative stress is considered as one of the mechanisms driving the age-related changes [167].

The level of oxidative stress in tissues is dependent on the balance between two separate mechanisms: 1) production of ROS (reactive oxygen species) and 2) the ability of antioxidant defense mechanisms to scavenge ROS. Yet, ROS are not only deleterious, but also essential signaling molecules needed for many adaptive processes and during development [168, 169]. Oxidative stress in the brain is thus not only related to the level of oxidative damage but also to the intriguing redox signaling, which can, for example, regulate growth of neural progenitor cells [170] and control axonal pathfinding and regeneration [171].

The production of ROS is directly linked to the metabolic activity of the cells, and specifically, the mitochondrial respiratory activity as mitochondria is the main source of ROS [172, 173]. Apart from mitochondria, also peroxisomes produce ROS as byproducts of their metabolic processes such as fatty acid oxidation [174]. Mitochondrial ROS are produced as a consequence of normal respiratory chain (RC) activity. While the normal respiratory function leads to relatively low ROS generation, RC defects may increase ROS production significantly (reviewed in Ref. [116]).

As mitochondria are major producers of ROS, they are also well equipped with defense mechanisms for ROS scavenging (reviewed in Ref. [118]).

### Oxidative stress in astrocytes

Astrocytes are glycolytic in their metabolism, to the extent that they survive without active respiratory chain [175]; thus, neurons have been considered as the major ROS producers in the brain. However, recent reports have shown that astrocytes can contribute to the redox status of the brain both directly by producing ROS and indirectly by affecting neuronal metabolism. Neurons rely on astrocytes for lactate supply and diminished lactate supply from astrocytes is likely to alter the oxidative microenvironment in the brain by increasing neuronal respiratory activity and reducing neurovascular coupling [176]. In addition, upon inflammation ROS is produced by both activated microglia and astrocytes to serve in the immune response and to trigger signaling cascades [177, 178]. Several antioxidant response pathway genes are regulated by the transcription factor Nrf2, which binds to the antioxidant response element (ARE) present in these genes. In normal physiological conditions, Keap1 targets Nrf2 for ubiquitin-mediated degradation; however, in oxidative conditions, Keap1 is not able to bind Nrf2 leading to accumulation of Nrf2 in the nucleus and induction of ARE-containing genes [179, 180]. This Nrf2–ARE pathway is particularly weak in neurons, whereas astrocytes both show strong response to Nrf2 activation and have higher expression of ARE-containing genes than neurons (Fig. 1e) [181]. Isolated neurons are known to possess very limited antioxidant defenses, including low catalase and GSH levels, both regulated by Nrf2, and Nrf2 deficiency does not affect their expression [182]. Astrocytes on the other hand are much more resistant to oxidative insults. They have high capacity for production and storage of GSH and oxidative stress increases GSH release from astrocytes. The GSH released from astrocytes is degraded and the cysteine-rich products are taken up by neurons to be used as building blocks for neuronal GSH synthesis in a process regulated by Nrf2 [183, 184]. Nrf2 deficiency also increases astrocyte vulnerability to oxidative stress [182].

Despite their low antioxidant defenses but high mitochondrial respiratory activity and consequently relatively high ROS production, post-mitotic neurons survive and remain functional for decades. This is thought to be achieved through antioxidant support from surrounding astrocytes [185].

### Alzheimer’s disease

Aβ plays a key role in oxidative stress induction in AD and astrocytes seem to be involved in this. Aβ exposure in neuron–astrocyte co-cultures leads to a loss of mitochondrial membrane potential and increased ROS production in astrocytes but not in neurons [186]. The increase in ROS is due to the activation of astrocytic NADPH oxidase, as inhibition of it blocks the ROS production and protects neurons [186]. The increased ROS due to activated NADPH oxidase further activates PARP, which leads to decreased NAD levels and metabolic failure in astrocytes further leading to neuronal death [187]. These data from rodent models strongly suggest that astrocytes have a central role in oxidative stress induction in AD. Human data are still scarce; however, our recent report from PSEN1 mutant patient astrocytes showed that also human AD patient astrocytes manifest Aβ deposits and altered metabolism with increased ROS production and these result in reduced neuronal support [188].

Oxidative stress directly affects Ca^2+^-homeostasis [189], which is one of the hallmarks of astrocytes and impaired in AD. In astrocytes, increased ROS production together with Ca^2+^ leakage from ER leads to opening of the mitochondrial permeability transition pore and transient depolarization of mitochondria, resulting in loading of cellular Ca^2+^ from ER to mitochondria [190]. The transient events were not detrimental; however, they eventually lead to complete loss of membrane potential and necrotic cell death and further increase in ROS production [190].

Astrocytic activation of transcription factor Nrf2 results in upregulation of antioxidant responses spreading to surrounded neurons [191, 192]. It has been demonstrated that lentiviral Nrf2 gene transfer causes a robust reduction in astrocytic activation and induction of Nrf2 target gene heme oxygenase 1 [193]. Finally, activation of the Nrf2 pathway by small molecules has been shown to confer protection in several neurodegenerative disorders, including AD and PD [194], and studies using mice with astrocyte-specific overexpression of Nrf2 prove that this neuronal protection is mediated by astrocytes, further signaling the importance of astrocytes in oxidative stress and neurodegeneration [184, 195].

### Amyotrophic lateral sclerosis

RC dysfunction in an SOD1 mutant rat model for ALS has been shown to lead to increased ROS production by astrocytes and motor neuron loss, which can be prevented by treating the astrocytes with antioxidants, suggesting a role for astrocytes in induction of oxidative stress also in ALS [196].

NF-κB transcription factors are central in inflammation and activated mainly by pro-inflammatory stimulation, which is present in many neurodegenerative diseases. However, also ROS has a strong role in regulating NF-κB signaling, and again, certain NF-κB-regulated genes are involved in regulating cellular ROS levels [197]. The most direct way for NF-κB to regulate cellular ROS levels is through activating antioxidant genes such as SOD2, SOD1, and thioredoxins [198–200]. However, as inflammation stimulates ROS production, NF-κB can activate also genes that lead to increased ROS. These include NADPH oxidase and inducible nitric oxide synthase (iNOS) [201, 202].

How ROS regulates NF-κB signaling in astrocytes is more complex. First of all, ROS has been reported to stimulate NF-κB in the cytoplasm, but inhibit it in the nucleus and many of the interactions of ROS and NF-κB are cell type and even astrocyte-specific [203]. Oxidation of cysteines is the most well characterized way that ROS affects NF-κB pathway. The initial reactions to sulfenic acid are usually reversible by antioxidants, but further oxidation leads to irreversible changes [203]. ROS can inhibit NF-κB DNA binding by direct oxidation of a cysteine of p50 [204]. The same cysteine can also be s-nitrosylated by NO produced by iNOS, which is activated by NF-κB, thus creating a negative feedback loop [205]. NF-κB activation may also further induce COX-2 and phospholipase A2 expression and promote inflammation [206].

## Modelling astrocytic dysfunction in human iPSC platforms

The majority of the current human data from neurodegenerative diseases originates from post-mortem samples, representing thus only the end stage of the disease. Novel methodologies have enabled the generation of comprehensive iPSC cell libraries [207], which will in the future be an invaluable model resource for studying neurodegeneration. For example, iPSC-based studies have shown aberrant morphological changes in AD astrocytes [208], as well as APOE-related neurotrophic and synaptic disturbances [19]. Our recent results further confirm the central role of astrocytes in AD and synaptic defects. We studied the consequences of mutant PSEN1 ΔE9 in patient iPSC-derived astrocytes and how it could be linked to AD pathology [188]. Our PSEN1 ΔE9 astrocytes demonstrated increased Aβ production and oxidative stress while showing reduced lactate production. Furthermore, the PSEN1 ΔE9 astrocytes influenced the Ca^2+^ signaling in healthy neurons. Both the glutamate and GABA-induced Ca^2+^ responses were reduced in neurons cultured with AD astrocytes in comparison with when the neurons were cultured with isogenic control astrocytes, suggesting that synaptic abnormalities indeed occur early in the disease course via astrocyte dysfunction. In addition, we were able to demonstrate that PSEN1 ΔE9 mutant astrocytes have altered cytokine secretion after pro-inflammatory stimulation [188].

In addition, the novel stem cell-based BBB models are very promising [209–211]. They have already resulted in advances in HD studies, showing that hiPSC-derived microvascular ECs had intrinsic abnormalities in angiogenesis and barrier properties [212]. In another recent study, a human BBB model based on hiPSCs from a patient carrying a mutation in transporter MCT8 revealed a decreased transport of l-triiodothyronine affecting neuronal function [213]. Studies to model BBB in AD, stroke, and traumatic brain injury are being developed [214]. hiPSC-based BBB models hold great promises for disease modeling and drug development and could provide new insights into how vascular dysfunction contributes to neurodegenerative diseases.

New iPSC techniques have also been exploited in ALS studies. Studies on ALS astrocytes of SOD1 mice and sALS patients as well in human ALS iPSC-derived astrocytes revealed elevated levels of connexin 43, which were associated with increased intracellular Ca^2+^ levels that regulate motor neuron survival [215]. One of the key roles of astrocytes is to support motoneurons, and importantly, a recent study discovered a neuronal help-me signal, which seems to be disrupted in ALS-patient-derived iPSC astrocytes and to lead to impaired STAT3 activation [118]. In this study, cell-autonomous mechanisms were found to diminish EphB1-ephrin-B1-mediated reverse signaling and STAT3 activation in ALS astrocytes [118]. The deficit was observed to cause a failure in maintenance of neuroprotective status that is normally induced by neuronal signals upon injury in healthy controls. Moreover, the detailed analysis of ALS astrocytes revealed an increased expression of PHLDA3, a regulator of Akt phosphorylation previously linked with cell death and degeneration in response to oxidative stress [216]. Thus, astrocytes in ALS and potentially in other neurodegenerative diseases may display loss-of-function type changes that could be valuable in future development of more efficient therapies.

The iPSC-based models revealing a central role of astrocyte dysfunctions in pathogenesis of neurodegenerative diseases makes them attractive target for drug discovery. Due to complexity of pathophysiological mechanisms, pre-clinical models that adequately reflect this are indispensable for the development of novel pharmacological treatments [217]. Currently, a number of in vitro models clearly indicate a pivotal role of astrocytes modulating neuronal responses, including their ability to survive environmental stress.

An important aspect on the drug discovery is the safety issues, particularly neurotoxicity in humans [218]. The induction of seizures is among the main adverse effects causing a failure of clinical trials. In a study, where hiPSC- derived neurons were co-cultured with astrocytes, activation of pathways related to AMPA and NMDA receptors, neuronal polarity, and axon guidance was demonstrated [219]. These results indicate that hiPSC-derived astrocytes promote neuronal susceptibility to convulsant drugs and that co-culture of hiPSC-derived neurons with hiPSC-derived astrocytes allows for a pre-clinical risk assessment of drug-induced seizures.

Astrocytes differentiated from human ESCs and iPSC have been used to screen compound libraries aiming at identification of molecules that could counteract oxidative stress, a common denominator in neurodegeneration [220]. Here, in a study by Ishii et al., a total of 4100 FDA approved drugs and compounds were screened and 9 of the 22 hits were validated in astrocytes differentiated from human iPSC. The protective mechanism proposed for the validated hits was implicating activation of antioxidant response though induction of Nrf2.

## Transplantation of glial progenitor cells and astrocytes

Animal models are used extensively to validate therapeutic targets, but confirmation of cellular and molecular mechanisms in human cells in vivo could increase the probability of successful target validation and drug development. Human glial chimeras may provide useful models for assessing the role of glia targets in human cognitive processing. The ability to construct the mice with glia generated from human ESCs and iPSCs allows the establishment of human glial chimeric mice with glia from defined disease genotypes and phenotypes. The capability promises to significantly advance our understanding of the contribution of glia to the pathogenesis of a wide variety of neurological disorders, many of which have simply been assumed to be entirely neuronal by origin.

Chimeric glial brains can be generated in vivo by a neonatal transplantation of human glial progenitors (A2B5+/PSA–NCAM cells obtained from 17- to 22-week-old human fetuses). Maturated recipient’s brains show large numbers and high proportions of both human glial progenitors and astrocytes, which retain specific hominid features despite of integration with the host astroglia and formation of gap junctions. The chimeric mice show increased performance in a number of behavioral tests such as maze navigation and fear conditioning [12], indicating the special features of human astrocytes and the value of such chimeric mouse models.

When human glial progenitor cells are transplanted into congenitally hypomyelinated shiverer mice, which have deficits in myelin generation and die by 18–21 weeks of age, the lethal phenotype is rescued and neurological performance improved [221]. The follow-up of 1 year after transplantation revealed massive expansion of human glia, outcompeting host cells [222].

Thus, the models based on disease-specific iPSC-derived human glial chimeras may provide an opportunity for better understanding of astrocytic dysfunction in a broad range of neurodegenerative and neuropsychiatric disorders [223].

A chimeric model comprising neonatal engraftment of human glial progenitor cells derived from mutant huntingtin transduced fetal hGPCs into immunodeficient mice has been established [224]. In these animals, striatal neurons are hyperexcitable and they display worse motor performance than controls. In contrast, striatal transplantation of normal glia into R6/2 HD mice counteracts the disease phenotype by restoration of interstitial potassium homeostasis, improving electrophysiological and behavioral test results, slowing down disease progression and extending the survival.

Recently, a human glia chimeric model was established by transplanting astroglia progenitors differentiated from iPSC lines of childhood-onset schizophrenic subjects [225]. These glial chimeras developed abnormal astrocytic morphology and hypomyelination compared to glial chimeras generated with cells derived from control iPSC lines. An impairment in the expression of differentiation-associated genes was evident and glial progenitors of schizophrenic subjects and corresponding chimeric mice demonstrated a broad range of behavioral and sleep abnormalities. Even though schizophrenia is not a neurodegenerative disease, this study shows further evidence for the importance of astrocytes in brain diseases.

The demonstrated non-cell-autonomous mechanisms in ALS provide a rationale for developing therapeutic approaches based on transplantation of glial progenitors. In a recent study human neural progenitor, cells engineered to produce GDNF were transplanted into the G93A SOD1 ALS rat cortex [226]. The transplanted cells maturated to astrocytes and released GDNF. The protection of motoneurons, delayed disease onset, and extended survival by transplanted GDNF producing progenitors were evident.

Spinal neural progenitor cells derived from healthy fALS and sALS iPSCs have been transplanted into cervical spinal cord of adult SCID mice [28]. Nine months later differentiated human astrocytes were found in large areas of the spinal cord, replacing host astrocytes and forming contacts with neurons. Importantly, both fALS and sALS cell transplantations caused reduction in motoneuron numbers and remaining motoneurons showed reduced inputs from inhibitory neurons and exhibited disorganized neurofilaments and aggregated ubiquitin. In addition, the cellular pathology in the spinal cord was accompanied with motor deficits. These results indicate that both fALS and sALS iPSC-derived astrocytes are capable to induce ALS-like pathological phenotype [227]. Importantly, transplantation into wt and ALS spinal cord showed no gross differences in the engraftment or gene expression in the differentiated human astrocytes, suggesting that the phenotype of transplanted glial progenitors is independent from the neurodegenerative environment supporting thus clinical transplantations into ALS patients [228].

Furthermore, HD patient-specific iPSCs were differentiated into neural progenitor cells and functional neurons in vitro [229]. When transplanted in vivo, they gave rise to neurons in the adult mouse brain. In parallel, astrocytes derived from HD iPSCs showed increased cytoplasmic vacuolation. Altogether, these results support feasibility to recapitulate HD phenotype under basal conditions what has been demonstrated in primary cells from HD patients.

In studies, where transplantation of glial-rich neural progenitors derived from human iPSCs was found to be beneficial, the potential therapeutic mechanism was assumed take place via activation of AKT signaling [230]. In one study, where primary glial-restricted precursor cells were transplanted focally into cervical spinal cord of a transgenic ALS rat, concomitant reduction of gliosis, extended survival, attenuated motor neuron loss and improved motor functions was observed to be partially mediated by astrocyte glutamate transporter GLT1 [231].

Human fetal-derived neural progenitor cells have been shown to survive well and mainly differentiate into astrocytes when transplanted into aging rat spinal cord [232]. Interestingly, differentiation into astrocytes was considerably more efficient in aged rats (500 and 600 days) than when using younger animals (100 and 200 days), possibly because aged environment may have more factors that stimulate astrocyte maturation compared to young adults. The differentiated astrocytes of the transplantation prevented the motoneuron loss that is associated with aging, even though improved motor performance was not evident.

One approach to obtain healthy, potentially therapeutic astrocytes into the CNS is injection of human mesenchymal stem cells (MSCs) into the cerebrospinal fluid. When symptomatic transgenic ALS rats received such injection, MSCs infiltrated into the nervous parenchyma and migrated substantially into the ventral gray matter [233]. Importantly, the injected MSCs efficiently differentiated into astrocytes, reduced motoneuron loss, and prolonged survival. The beneficial effect of human MSC-derived astrocytes was possibly mediated by reduced inflammation, since the neuroprotection correlated with the inhibition of microglial proliferation and reduction of COX-2 and NOX-2 expressions.

Transplantation of precursor cell-derived astrocytes has also been studies in a rat model of PD. The astrocytes were generated in vitro by directed differentiation of glial precursors. The transplantation into striatum of the 6-hydroxydopamine lesioned rat model restored HT expression rescued GABAergic interneurons and improved behavioral recovery [234]. It is thought that transplanted cells produced beneficial trophic factors and antioxidants. Further studies have explored cografting astrocytes, derived from the midbrain and demonstrated that they remarkably enhanced neural progenitor cell differentiation into maturated DA neurons and promoted DA neuron engraftment in PD rats for at least 6 months after transplantation [235].

A number of studies have investigated astrocyte transplantation in AD models. When astrocytes isolated from adult and neonatal mice were transplanted into the hippocampi of AD model APdE9 mice most of them were found near Aβ deposits and internalized human Aβ immunoreactive material [236]. In the follow-up study, a significant reduction in Aβ burden was observed after 2 months in parallel colocalization with transplanted astrocytes, and the remaining Aβ deposits showed fragmentation [237]. The mechanism demonstrated was associated with astrocytic expression of Aβ degrading proteases such as neprilysin (NEP), angiotensin-converting enzyme-1 (ACE-1), and endothelin-converting enzyme-2 (ECE-2).

Another approach by autologous transplantation of glia cells isolated from intestine—enteric glial cells (EGCs) has demonstrated an improvement in neuropathology and restoration of cognitive deficits in of a rat model of Aβ-induced neurodegeneration [238].

## Conclusions

Astrocytes have been shown to take active part in nearly all CNS functions and participate both in initiation and progression of neurodegenerative disease. As human astrocytes are much more complex than their rodent counterparts, human models are needed to be able to better understand the role of astrocytes in human neurological disease. Moreover, there is strong evidence, indicating that activation of microglia precedes the pro-inflammatory activation of astrocytes in rodents. Considering that inflammation is involved in aging and most of the brain diseases, models including both human astrocytes and human microglia that interact with human neurons are going to further reveal the multiple functions of human astrocytes. Thus, the novel human stem cell-based models are indispensable to discover pathological mechanism and for development of therapeutic strategies.

## Abbreviations

AD: Alzheimer’s disease
ALS: Amyotrophic lateral sclerosis
APP: Amyloid precursor protein
ARE: Antioxidant response element
BBB: Blood–brain barrier
CNS: Central nervous system
EC: Endothelial cells
ES: Embryonic stem
GDNF: Glial cell line-derived neurotrophic factor
MERTK: Mer proto-oncogene tyrosine kinase
MS: Multiple sclerosis
PD: Parkinson’s disease
PET: Positron emission topography
PPP: Pentose phosphate pathway
RC: Respiratory chain
RET: Reverse electron transport
ROS: Reactive oxygen species
VEGF: Vascular endothelial growth factor
